# Ultrasound-Guided Microwave Ablation of Thyroid Schwannoma

**DOI:** 10.1210/jcemcr/luae146

**Published:** 2024-08-12

**Authors:** Xue Han, Yuzhi Zhang, Yu Li, Ruiping Li, Chao Liu, Shuhang Xu

**Affiliations:** Endocrine and Diabetes Center, The Affiliated Hospital of Integrated Traditional Chinese and Western Medicine, Nanjing University of Chinese Medicine, Nanjing 210028, China; Department of Ultrasound, The Affiliated Hospital of Integrated Traditional Chinese and Western Medicine, Nanjing University of Chinese Medicine, Nanjing 210028, China; Department of Radiology, The Affiliated Hospital of Integrated Traditional Chinese and Western Medicine, Nanjing University of Chinese Medicine, Nanjing 210028, China; Department of Pathology, The Affiliated Hospital of Integrated Traditional Chinese and Western Medicine, Nanjing University of Chinese Medicine, Nanjing 210028, China; Endocrine and Diabetes Center, The Affiliated Hospital of Integrated Traditional Chinese and Western Medicine, Nanjing University of Chinese Medicine, Nanjing 210028, China; Key Laboratory of Traditional Chinese Medicine Syndrome and Treatment of Yingbing (Thyroid Disease) of State Administration of Traditional Chinese Medicine, Jiangsu Province Academy of Traditional Chinese Medicine, Nanjing 210028, China; Endocrine and Diabetes Center, The Affiliated Hospital of Integrated Traditional Chinese and Western Medicine, Nanjing University of Chinese Medicine, Nanjing 210028, China

**Keywords:** microwave ablation, thyroid schwannoma, core needle biopsy, volume reduction

## Abstract

Thyroid schwannoma, a rare neoplasm of the thyroid gland, originates from Schwann cells that form the myelin sheath. A 47-year-old woman presented with a progressively enlarging thyroid nodule, which was monitored by repeated ultrasonography over the previous 2 years. Following a diagnosis of thyroid schwannoma by core needle biopsy and immunohistochemical staining, the patient underwent ultrasound-guided microwave ablation (MWA). Subsequent thyroid ultrasounds indicated a gradual decrease in the tumor's volume, achieving a 12-month volume reduction ratio of 79.20%. No complications were observed. Ultrasound-guided MWA may serve as an effective alternative to conventional surgery for managing thyroid schwannomas.

## Introduction

Schwannoma, a benign and well-encapsulated tumor of the nerve sheath, consists exclusively of Schwann cells originating from neural crest cells (1, 2). The World Health Organization recognizes schwannoma as a grade I benign tumor. Schwannomas arising in the thyroid are notably rare. Diagnoses through fine-needle aspiration cytology (FNAC) or imaging pose considerable challenges. Historically, surgical resection has been the preferred treatment strategy for thyroid schwannoma. Conversely, thermal ablation, a less invasive approach, has seen broad application in managing both benign and malignant thyroid nodules (3, 4). Moreover, recent reports have documented the safe and effective use of MWA for treating extracranial schwannomas of the cervical vagus nerve in the carotid space (5). This study discusses a case of thyroid schwannoma managed with ultrasound-guided MWA and reviews related clinical literature.

## Case Presentation

During a routine physical, a thyroid nodule was identified in the left lobe of a 47-year-old woman; it had noticeably enlarged over the last 2 years. She reported a mild foreign body sensation in her neck. Symptoms such as hoarseness, dysphagia, cough, and neck pain were absent. A grade 2 goiter was noted, and a firm, movable thyroid nodule measuring 2.5 cm was palpable in the left lobe.

## Diagnostic Assessment

**Figure 1.**
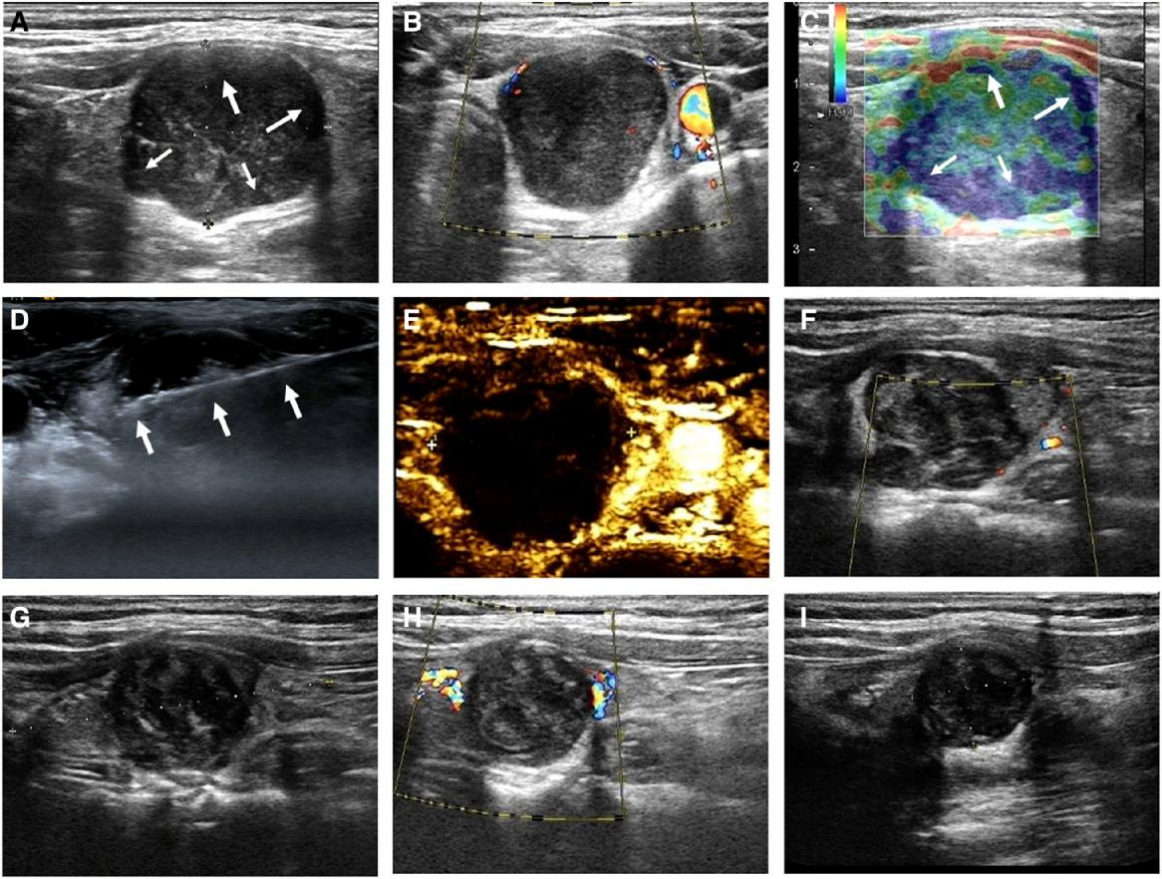
Representative preoperative, intraoperative, and postoperative ultrasound scans of the thyroid. A, Preoperative gray-scale ultrasonography of the thyroid showing a heterogeneous hypoechoic nodule in the left lobe. B, Color Doppler ultrasound of the thyroid indicating less vascularity at the margin of the thyroid nodule. C, Elastography showing the target sign (white arrows) of the thyroid nodule with central soft tissues (green) and surrounding hard tissues (blue). D, Intraoperative ultrasonography depicting the microwave antenna (white arrows) inserted into the thyroid nodule. E, Ultrasonography the day after microwave ablation showing the absence of perfusion in the ablation zone. F to I, Ultrasonography at F, 1; G, 3; and I, 12 months postoperatively demonstrating a gradual reduction in tumor volume.

Gray-scale ultrasonography identified a solid, clearly bounded, regularly shaped hypoechoic nodule in the left thyroid gland, measuring 2.78 cm × 2.16 cm × 2.15 cm. This nodule was classified as Chinese Thyroid Imaging Reporting and Date System (C-TIRADS) 4A (Fig. 1A) and as Adler grade 1 on the color Doppler flow ultrasound image (Fig. 1B). Elastography detected a moderately stiff thyroid nodule with soft tissues at the center and hard tissues at the margin (Fig. 1C). All thyroid function indices, hormones, and related laboratory parameters were within normal limits. Specifically, serum free triiodothyronine was 4.51 pmol/L (0.29 ng/dL), free thyroxine 15.02 pmol/L (1.17 ng/dL), and thyrotropin 2.39 μIU/mL (2.39 mIU/L). Levels of thyroglobulin antibody, thyroid peroxidase antibody, thyroglobulin, parathyroid hormone, calcium, calcitonin, and carcinoembryonic antigen (CEA) also remained within normal ranges.

**Figure 2.**
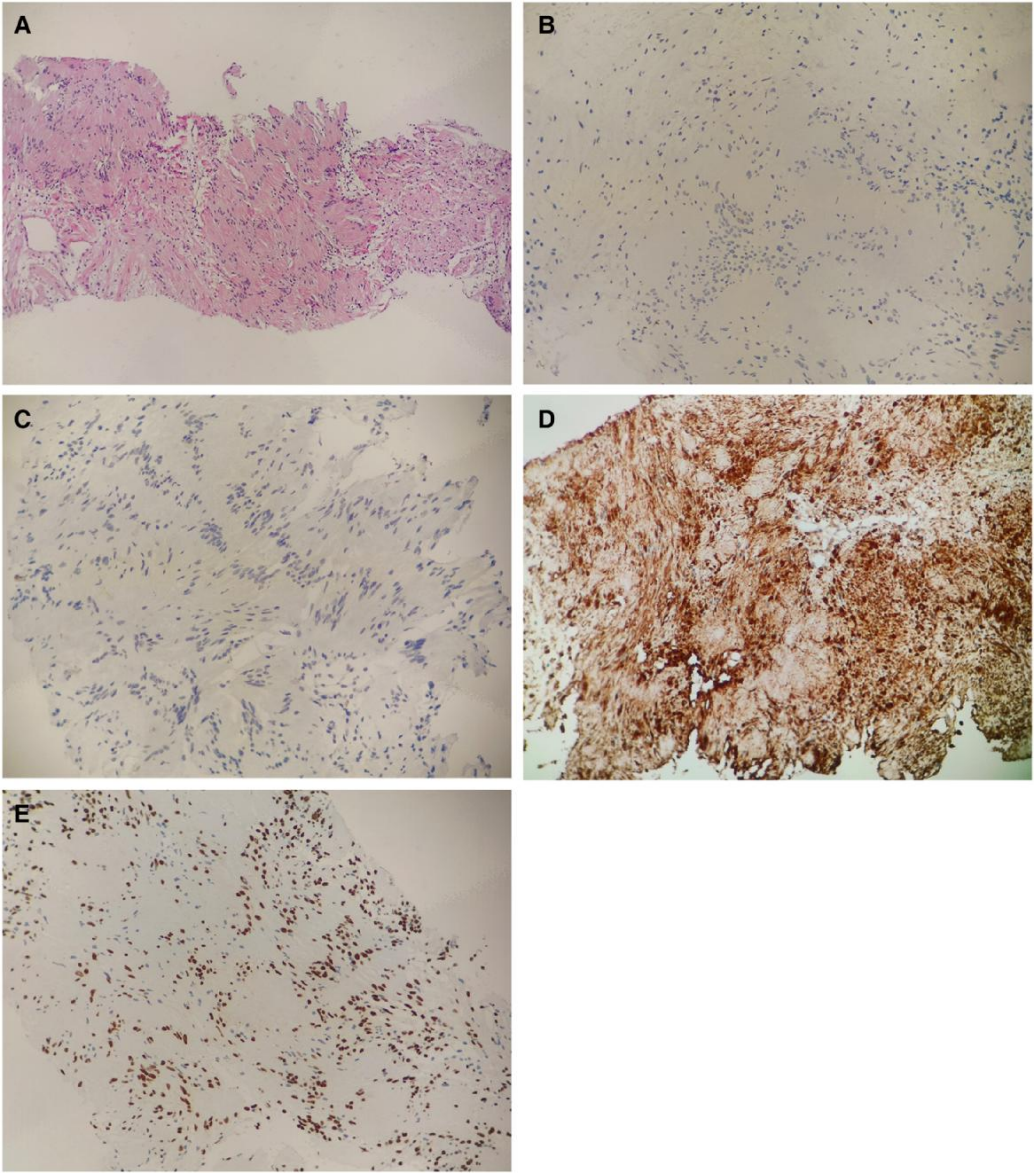
Histopathology and immunochemistry staining of the thyroid schwannoma. A, Immunohistochemical staining on thyroid lesions collected via core needle biopsy (magnification = 100×). B, Immunohistochemical staining for TTF1; C, PAX8; D, S100; and E, SOX10. Magnification = 200×.

FNAC showed inadequate cellularity, and common gene mutations associated with thyroid carcinomas were absent, including *BRAFV600E*, *RAS*, *PTEN*, *ALK*, *NTRK*, *EIF1AX*, and *DICER1*. CNB and immunohistochemical staining were subsequently conducted to establish a precise diagnosis. The thyroid nodule tested positive for TTF-1 (in partial cells), galectin-3, and Ki-67 (approximately 3%), as well as CD56, S100, and SOX-10, but negative for PAX-8, HBME-1, BRAF (VE1), CT, CEA, CK19, and CKpan, confirming a diagnosis of schwannoma (Fig. 2). Computed tomography of the neck showed a low-density thyroid nodule in the left lobe, measuring 1.9 cm × 2.0 cm, with no lymph node abnormalities.

## Treatment

Ultrasound-guided MWA was implemented following a multidisciplinary consultation. The patient was fully informed about the potential efficacy and risks. The MWA procedure for the thyroid schwannoma was performed under ultrasonographic guidance (Fig. 1D). During the 35-W point-by-point ablation, normal saline was continuously injected to serve as an isolating fluid, preventing heat damage to adjacent normal tissues. The ablation zone was extended 2 to 5 mm beyond the thyroid nodule's capsule. After confirming the absence of blood flow within the thyroid nodule via ultrasonography, the ablation catheter was removed. The total ablation time lasted 5 minutes and 34 seconds. A total of 39 mL of normal saline was injected intraoperatively to create and maintain a liquid barrier, safeguarding surrounding structures. The patient reported only mild pain during the procedure.

## Outcome and Follow-up

**Figure 3.**
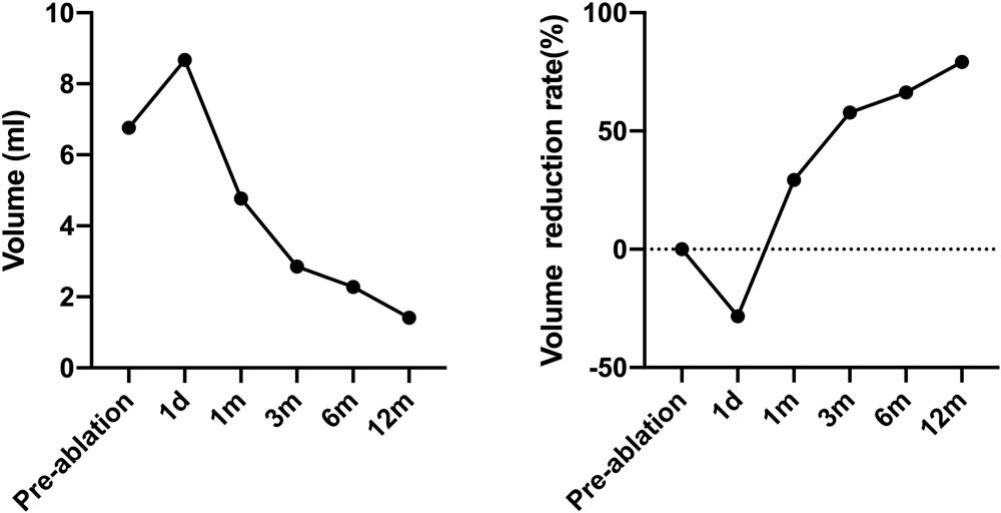
Changes in the volume (left) and volume reduction rate (right) of the thyroid schwannoma.

Postoperative ultrasonography confirmed the absence of blood flow within the thyroid nodule and any residual residues. The volume of the postoperative thyroid schwannoma measured 8.67 mL (3.37 cm × 1.99 cm × 2.47 cm), representing a 28.30% increase over the preoperative volume (Fig. 1E). Postoperative thyroid function and electrolyte levels were normal. Subsequent thyroid ultrasounds indicated volume reduction rates (VRRs) of 29.36% at 1 month, 57.87% at 3 months, 66.30% at 6 months, and 79.20% at 12 months, demonstrating a satisfactory outcome of the MWA (Fig. 1F-1I, Fig. 3). No adverse events such as hoarseness or local pain were reported.

## Discussion

Schwannoma is benign, composed of Schwann cells from the neuroectoderm, and occurs solitarily in more than 90% of cases (6). In approximately 90% of instances, schwannomas originate from the vestibular nerve. However, they may also arise from the facial, auditory, and trigeminal nerves (7). Nonepithelial tumors of the thyroid, such as lipomas, lymphomas, teratomas, hemangiomas, and schwannomas, are uncommon, comprising only 1% of all thyroid tumors (7). Primary thyroid schwannoma develops from intrathyroidal sensory, sympathetic, or parasympathetic nerves (8). The clinical presentation of thyroid schwannoma, typically encapsulated, benign, and isolated in the unilateral lobe, is nonspecific. Symptoms and signs are generally due to the compression of adjacent tissue. Diagnosis is predominantly made through histopathology after surgical resection. Preoperative blood tests and radiographic studies typically reveal no specific abnormalities.

On ultrasound scans, thyroid schwannoma typically appears as oval-shaped, well-defined, hypoechoic, and heterogeneous nodules with reduced intranodular vascularity; cystic components may also be present (9, 10). However, these ultrasonographic features are similar to those of goiter and adenoma, posing considerable challenges for differential diagnosis. Unlike schwannomas arising from peripheral nerves, primary thyroid schwannomas do not exhibit the “rat tail” sign typically seen on ultrasonography. Yang et al (11) reported that the “target sign” is observable in 44.4% of cases involving schwannomas from peripheral nerves on ultrasound scans, and it is also characteristic on magnetic resonance imaging. Similarly, Yuan et al (10) found the “target sign” in 82.5% of such cases on elastography. Therefore, the “target sign” may serve as a distinguishing ultrasonographic feature for identifying thyroid schwannoma from other thyroid nodules. Schwannomas exhibit moderate stiffness on elastography, which suggests benignity (9, 10). Notably, the “target sign” is more pronounced on elastography, making it an effective tool for differentiating thyroid schwannoma from thyroid carcinoma (10). The use of contrast-enhanced ultrasound (CEUS) in examining thyroid schwannoma has been infrequently reported, typically showing heterogeneous hypoenhancement (9). In this case, consistent imaging characteristics were noted, including hypoechoic regions near and at the margins of the tumor with poor blood flow on the ultrasound, and a pronounced “target sign” on elastography (see Fig. 1A).

Thyroid schwannoma is typically diagnosed post thyroidectomy, as FNAC often fails to provide a precise preoperative diagnosis. This issue may stem from improper sampling (12). Due to the nonhomogeneous nature of the lesions, up to 80% to 90% of thyroid schwannoma cases involve inadequate tissue sampling. CEUS has been demonstrated to improve the success rate of percutaneous needle biopsies (12‐14). In FNAC, guided by CEUS, tumor cells can be aspirated from notably contrast-enhanced areas, yielding highly viable specimens (9). In this case, FNAC yielded inadequate cellularity, but the diagnosis of thyroid schwannoma was confirmed through CNB and immunohistochemical staining. Histologically, thyroid schwannoma is characterized by Antoni A and Antoni B areas, both of which are immunoreactive for S-100 and vimentin. In our case report, immunohistochemical staining revealed positive expressions for TTF-1 (in partial cells), galectin-3, Ki-67 (approximately 3%), CD56, S100, and SOX-10, and negative expressions for PAX-8, HBME-1, BRAF (VE1), CT, CEA, CK19, and CKpan.

Follow-up, surgery, and radiotherapy are standard approaches to managing schwannomas (6). Although surgical resection can lead to substantial physical deficits, regular follow-up is advised for patients with benign schwannomas (15). Schwannomas grow slowly, compressing rather than invading surrounding tissues. Thus, dissection and marginal resection of thyroid schwannoma do not harm adjacent nerves. The prognosis for schwannoma is generally favorable, depending on the tumor's size, location, and the patient's physical condition (16). Reports of postoperative recurrence are rare. Malignant schwannoma is uncommon and usually occurs in patients with neurofibromatosis. Radiotherapy is generally not recommended due to the tumor's insensitivity to this treatment modality.

Thermal ablation, including MWA, radiofrequency ablation, laser ablation, and high-intensity focused ultrasound, is frequently employed for treating thyroid nodules. Currently, guidelines have been established to standardize thermal ablation for benign thyroid nodules, microcarcinomas, and cervical metastatic lymph nodes (3, 4, 17, 18). Efficacy is assessed primarily through imaging (with a preference for elastography), VRR at 3, 6, and 12 months, clinical outcomes, and postoperative pathology (4). Previous data indicate that MWA is safe and effective for benign thyroid nodules and low-risk papillary thyroid microcarcinoma (19, 20). However, thermal ablation has not been previously applied to thyroid schwannoma. In 2022, Zhu et al (5) treated a cervical schwannoma in a 65-year-old woman with a history of non–small cell lung cancer using ultrasound-guided MWA under general anesthesia. The tumor continued to shrink postoperatively. In the present case, a 12-month VRR of 79.20%, normal postoperative thyroid function, and the absence of reported complications or adverse events all confirm the high efficacy and safety of ultrasound-guided MWA for thyroid schwannoma.

Taken together, the present case of thyroid schwannoma was ultimately diagnosed through CNB and immunohistochemical staining, following the ineffectiveness of FNAC and molecular testing. Ultrasound-guided thermal ablation represents an emerging therapeutic alternative to conventional surgery for thyroid nodules. Future studies are required to validate the long-term efficacy and safety of ultrasound-guided MWA for thyroid schwannomas in a large cohort.

## Learning Points

- Primary thyroid schwannoma is extremely rare and is seldom diagnosed through FNAC or preoperative imaging examinations; however, it can be identified via CNB and immunohistochemical staining.
- The “target sign” on elastography may act as a distinctive ultrasonographic indicator for differentiating thyroid schwannoma from other thyroid nodules.
- Surgical resection is the treatment of choice for thyroid schwannoma, and the postoperative pathology invariably indicates benignity.
- As an emerging therapeutic option, ultrasound-guided thermal ablation offers a viable alternative to traditional surgery for benign thyroid nodules.

## Contributors

All authors made significant contributions to the manuscript. X.H. and S.X. conceptualized and designed the case report, gathered, and analyzed the data, and drafted the initial and final manuscript. Y.Z., Y.L., R.L., and C.L. contributed to the diagnosis and management of this patient. All authors reviewed, revised, and approved the final manuscript as submitted and agree to be accountable for all aspects of the work.

## Data Availability

Data sharing is not applicable to this article as no data sets were generated or analyzed during the current study.

## Abbreviations

CEA: carcinoembryonic antigen
CEUS: contrast-enhanced ultrasound
CNB: core needle biopsy
FNAC: fine-needle aspiration cytology
MWA: microwave ablation
VRR: volume reduction ratio
